# Experience with Linezolid for the Treatment of Rifampin-Susceptible Tuberculosis in San Francisco

**DOI:** 10.1093/ofid/ofaf714

**Published:** 2025-11-26

**Authors:** Janice K Louie, Gustavo E Velásquez, Allison Phillips, John D Szumowski

**Affiliations:** Tuberculosis Prevention and Control Program, San Francisco Department of Public Health, San Francisco, California, USA; Department of Medicine, University of California, San Francisco, San Francisco, California, USA; Division of HIV, Infectious Diseases, and Global Medicine, University of California, San Francisco, San Francisco, California, USA; UCSF Center for Tuberculosis, Institute for Global Health Sciences, University of California, San Francisco, San Francisco, California, USA; Tuberculosis Prevention and Control Program, San Francisco Department of Public Health, San Francisco, California, USA; Tuberculosis Prevention and Control Program, San Francisco Department of Public Health, San Francisco, California, USA; Department of Medicine, University of California, San Francisco, San Francisco, California, USA

**Keywords:** adverse events, drug-susceptible, linezolid, oxazolidinone, tuberculosis

## Abstract

Linezolid is recommended for the treatment of rifampin-resistant tuberculosis (TB), but its role in rifampin-susceptible TB (RS-TB) is less understood. In 45 RS-TB patients treated with linezolid, 8 stopped due to adverse events, most commonly cytopenias. Further research is needed on linezolid efficacy, dosing, and adverse event management in RS-TB.

## Teaser

With linezolid now part of first-line treatment recommendations (in combination with bedaquiline and pretonamid) for rifampin-resistant tuberculosis, there has been increasing interest in using linezolid for rifampin-susceptible TB (RS-TB); linezolid use for RS-TB in San Francisco increased from 3% in 2016 to 16.5% in 2024. With over 80% (38/45) receiving linezolid for a median duration of 91 days (range 1-365), we found that linezolid can be a useful component of RS-TB treatment regimens when using strategies including vigilant clinical monitoring for adverse events, therapeutic drug monitoring, and dose adjustment as needed.

Recent studies have highlighted the use of new or repurposed drugs for the treatment of multidrug-resistant/rifampin-resistant (MDR/RR) tuberculosis (TB) [1–6]. Linezolid is an oxazolidinone antibacterial drug used for the treatment of certain gram-positive bacterial infections and can cause mitochondrial-related toxicity, resulting in adverse events (AE) such as myelosuppression, peripheral neuropathy and optic neuritis [7]. However, linezolid remains appealing for use in TB treatment given its excellent anti-tuberculosis activity and properties such as good central nervous system and bone penetration, low risk of hepatotoxicity and available intravenous formulation [8]. It is now incorporated in recommended regimens for MDR/RR-TB [9–11]. Here, we describe our experience with linezolid for the treatment of rifampin-susceptible TB (RS-TB) at the San Francisco Department of Public Health (SFDPH) TB Clinic, which manages all patients with active TB residing in San Francisco.

## METHODS

We reviewed medical records of those with RS-TB from 1 January 2016 to 31 December 2024. Persons initiated on TB treatment underwent clinical, radiographic and microbiologic evaluation consistent with national recommendations [12]. Linezolid was used at the discretion of the treating clinician and initiated at 600 mg daily. When staffing and patient availability allowed, a serum trough concentration was obtained; specimens were sent to National Jewish Health (Denver, CO) or the University of Florida (Gainesville, FL). All patients had monthly screening for symptoms suggestive of linezolid AEs such as new numbness, tingling, or decreased sensation; if present, patients underwent serial monofilament exams. Monthly vision screenings included Snellen and Ishihara exams and measurement of complete blood count and liver function tests. The National Cancer Institute Common Terminology Criteria for Adverse Events were used to assess drug-associated AEs [13]. When indicated, the dose of linezolid was either reduced to 300 mg daily, 600 mg thrice weekly, or stopped depending on provider discretion; reasons included elevated linezolid trough levels >2 μg/mL or a new or worsening AE.

### Patient Consent Statement

The analysis of routinely collected SFDPH TB Clinic data was reviewed and approved by the Human Subjects Review Committee of the University of California, San Francisco (IRB #21-34264). A waiver of informed consent was granted for this retrospective analysis.

## RESULTS

**Figure 1.**
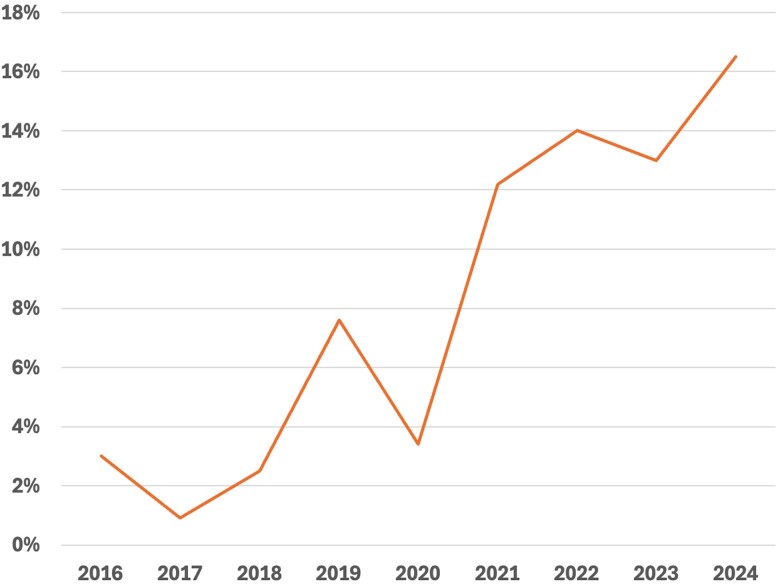
Trend in Linezolid Use for Treatment of Drug-Susceptible Tuberculosis in San Francisco, 2016–2024.

From 2016 to 2024, 773 San Francisco residents with RS-TB were treated, 56 (7.2%) of whom received linezolid as part of their TB treatment. The proportion of persons who received linezolid increased from 3% in 2016% to 16.5% in 2024 (Figure 1).

As of 1 June 2025, 45 patients have completed TB treatment (Table 1). The median age was 71 years (range 24–86), 31 (68.9%) were male and 33 (73.3%) had co-morbid illness(es). Most patients had pulmonary TB (23; 51.1%). Clinical indications for linezolid included AE(s) to a first-line drug (33; 73.3%) including drug-induced liver injury (23; 51.1%), and rifampin-related (9; 20.0%) or fluoroquinolone-related (8; 17.8%) AEs. Other reasons included isoniazid or fluoroquinolone resistance (12; 26.7%), and rifamycin drug-drug interaction (5; 11.1%). The most common anti-tuberculosis regimens included (1) liver-sparing combinations of linezolid, ethambutol, and a fluoroquinolone with (6; 13.3%) or without (6; 13.3%) a rifamycin and (2) combinations of linezolid, ethambutol, a fluoroquinolone, and isoniazid in patients who had either an AE or contraindication to rifamycins (8; 17.7%). The median linezolid treatment duration was 91 days for both immunocompetent and immunocompromised persons (range 1–379 and 58–365 days respectively). In total 19/45 persons (42.2%) had linezolid trough levels checked; of these, 5/19 (26.3%) had their linezolid dose reduced due to elevated trough level. Six of these 19 (31.6%) had linezolid-associated AEs; only one had a high trough.

**Table 1.** Characteristics of Patients Treated With Linezolid for Drug-Susceptible Tuberculosis in San Francisco, 2016–2024

Characteristic	Total (*N* = 45)
**Median age (range), years**	71 (24–86)
**Sex**	
Female	14 (31.1)
Male	31 (68.9)
**Comorbid illness** ^a^	
Previously healthy	12 (26.7)
Immunocompromised^b^	10 (22.2)
Chronic cardiac disease	9 (20.0)
Diabetes mellitus	8 (17.7)
Chronic kidney disease	6 (13.3)
Chronic liver disease	5 (11.1)
Chronic lung disease	5 (11.1)
Other^c^	15 (33.3)
**Site of Disease** ^d^	
Pulmonary	23 (51.1)
Gastrointestinal	4 (8.9)
Central nervous system	1 (2.2)
Pleural	1 (2.2)
Spine	1 (2.2)
Disseminated (pulmonary and extrapulmonary)	15 (33.3)
**Linezolid indication^a^**	
Adverse event to a TB drug	33 (73.3)
DILI to a first-line drug^e^	23 (51.1)
Adverse event to a rifamycin^f^	9 (20.0)
Adverse event to a fluoroquinolone^g^	8 (17.8)
Isoniazid resistance	10 (22.2)
Rifamycin drug-drug interaction	5 (11.1)
Chronic liver disease/need for liver-sparing regimen	4 (8.9)
Intravenous TB medications due to malabsorption	4 (8.9)
Contraindication to fluoroquinolone use^h^	4 (8.9)
Fluoroquinolone resistance	2 (4.4)
TB meningitis treatment	2 (4.4)
**Linezolid-containing regimen prescribed**	
Linezolid, ethambutol, and fluoroquinolone	6 (13.3)
Linezolid, ethambutol, fluoroquinolone, and isoniazid,	8 (17.7)
Linezolid, ethambutol, fluoroquinolone, and rifamycin	6 (13.3)
Linezolid, ethambutol, fluoroquinolone, isoniazid, rifamycin, and pyrazinamide	3 (6.7)
Linezolid, levofloxacin, and rifampin (all intravenous)	3 (6.7)
Other	19 (42.2)
**Linezolid-related adverse event^i^**	11 (24.4)
Grade 1 adverse event	1 (2.2)
Thrombocytopenia	1 (2.2)
Grade 2 adverse event	6 (13.3)
Anemia	1 (2.2)
Leukopenia	1 (2.2)
Thrombocytopenia	1 (2.2)
Other (glossitis)	1 (2.2)
Other (gastrointestinal upset)	1 (2.2)
Other (rash)	1 (2.2)
Grade 3 adverse event	4 (8.9)
Anemia	2 (4.4)
Leukopenia	1 (2.2)
Other (lactic acidosis)	1 (2.2)
Grade 4 or 5 adverse event	0 (0)
**Linezolid dose decreased due to adverse event^j^**	8 (17.8)
**Linezolid discontinued due to adverse event**	7 (15.6)
Thrombocytopenia	3 (6.7)
Glossitis	1 (2.2)
Lactic acidosis	1 (2.2)
Gastrointestinal upset	1 (2.2)
Rash	1 (2.2)
**Linezolid trough level collected**	19 (42.2)
Trough level collected during 600 mg daily dosing	15/19 (78.9)
Median linezolid trough level (range), ug/ml	1.60 (0–6.4)
Trough level collected during 600 mg three times/week dosing	4/19 (21.1)
Median linezolid trough level (range), μg/mL	0.42 (0–1.1)
Patients with elevated linezolid trough level (>2 μg/mL) leading to linezolid dose decrease	5/19 (26.3)
**Treatment durations**	
Median TB treatment duration (range), days	308 (4–596)
Median time on TB treatment before linezolid started (range), days	46 (0–366)
Median linezolid treatment duration (range), days	91 (1–379)
Patients who received ≥60 doses of linezolid	29 (64.4)
**Treatment outcomes**	
Treatment completed	36 (80.0)
Moved to another jurisdiction to complete treatment	1 (2.2)
Disease recurrence^k^	0 (0)
Death^l^	8 (17.8)

Values shown represent number (percent) unless otherwise specified.

Abbreviations: CTCAE, Common Terminology Criteria for Adverse Events; DILI, drug induced liver injury; MDR, multidrug-resistant; TB, tuberculosis.

^a^One or more characteristic may apply to an individual patient.

^b^Immunocompromising conditions included status-post organ transplant (2), acute myeloid leukemia status-post bone marrow transplant (1), acquired immunodeficiency syndrome (1), anti-tumor necrosis factor therapy (1), chronic leukopenia (1) and active chemotherapy for hepatocellular cancer (1), adenocarcinoma (1), lymphoma (1) and esophageal cancer (1).

^c^Other included hypertension (15), alcohol/substance use disorder (3), dementia (1), Crohn's disease (1), idiopathic thrombocytopenic purpura (1), prostate cancer (1), status-post transient ischemic attack (1), and history of prior malignancy [bladder (1), thyroid (1), gastric (1) and colon (1)].

^d^Sites of TB disease were classified according to criteria defined by the CDC Report of Verified Cases of TB definitions [14]. For pulmonary sites, this was defined as TB disease inside the lung structure, including lung parenchyma abnormalities identified on chest radiograph and/or by respiratory specimen positive by laboratory testing for TB. For pleural sites, this was defined as TB disease in the pleural space outside the lungs, including pleural effusion or thickening identified on chest radiograph and/or pleural fluid specimen positive by laboratory testing for TB.

^e^DILI attributed to any first-line drug including isoniazid, a rifamycin, or pyrazinamide.

^f^Rifamycin-related adverse events included rash (6), dizziness (1), nausea (1), thrombocytopenia (1), hyperbilirubinemia (1).

^g^Fluoroquinolone (levofloxacin or moxifloxacin) related adverse events included tendinitis (2), rash (2), joint pain (1), myalgias (1), nausea (1), dizziness (1), anorexia (1), headache (1).

^h^Fluoroquinolone contraindicated due to baseline prolonged QT interval (3) and vascular aneurysm (1).

^i^All adverse events were graded on a scale of 1–5 according to the CTCAE scale [2]. The table notes the highest grade adverse event diagnosed for an individual patient. No patients were diagnosed with a grade 4 (life-threatening consequences; urgent intervention indicated), or grade 5 (“death”) adverse event. CTCAE grade 1, 2 and 3 adverse events were recorded for anemia (Hgb decrease to lower limit of normal [LLN] −10 g/dL, < 10–8 g/dL and <8 g/dL, respectively), thrombocytopenia (platelets decrease to LLN-75,000/mm^3^, < 75 000–50 000 mm^3^, and <50,000- 25 000 mm^3^, respectively) and leukopenia (<LLN—3000 mm^3^, < 3000–2000mm^3^ and <2000–1000 mm^3^, respectively).

^j^Linezolid dose reductions were based on provider discretion, to decreased doses of either 300 mg daily or 600 mg three times a week.

^k^Disease recurrence was defined as clinical or bacteriologic diagnosis of TB disease up to 24 m after initiation of TB treatment.

^l^The cause of death on the patient's death certificate and upon medical record review was listed as malignancy (2), pulmonary TB (1), TB meningitis (1), sepsis (1), renal failure (1), heart failure (1), and respiratory failure (1). There was no documentation of deaths due to TB medication toxicity.

Linezolid-associated AEs were identified in 11/45 persons (24.4%); cytopenias were the most commonly reported AE (7/11, 63.6%). Eight of these 11 individuals (72.7%) had their linezolid dose decreased, with five showing subsequent AE improvement and three ultimately stopping linezolid. In total 7/45 persons (15.6%) stopped linezolid altogether due to AEs; of these six had linezolid trough levels collected, with one having high trough level necessitating downward dose adjustment before linezolid was ultimately stopped. Of the 10 patients with immunocompromising conditions, two required dose reduction and two required linezolid cessation (three due to AEs and one due to high trough level). No patients reported symptoms concerning for peripheral neuropathy.

Treatment outcomes included 36 (80%) treatment completions and eight (17.8%) deaths during treatment. The median duration of TB treatment was 308 days (range 4–596). In two of the eight deaths, the cause of death was considered TB-related.

## DISCUSSION

Studies over the last decade have highlighted the role of linezolid for treatment of MDR/RR-TB. The Nix-TB study found that a 6-to-9 month regimen of bedaquiline, pretomanid, and linezolid (BPaL) using linezolid 1200 mg daily was associated with a favorable outcome in 89% of participants, although linezolid-associated peripheral neuropathy and myelosuppression were common (81% and 48%, respectively) [1]. The ZeNix trial conducted dose- and duration-ranging studies of linezolid within BPaL (ranging from 600 to 1200 mg daily for 2–6 months) with similar treatment success; reduced doses and duration of linezolid were associated with fewer AEs [2]. Subsequently, the TB-PRACTECAL trial demonstrated that a 6-month regimen of BPaL plus moxifloxacin (BPaLM) (linezolid dosed at 600 mg daily for 16 weeks followed by 300 mg daily for 8 weeks) was non-inferior to standard MDR/RR-TB therapy, with rates of peripheral neuropathy and cytopenias of 9% and 1–4%, respectively [3]. BPaLM is now the preferred treatment regimen for fluoroquinolone-susceptible MDR/RR-TB [10, 11, 15]. Recent clinical trials for MDR/RR-TB have further studied regimens that incorporate starting linezolid doses at 600 mg daily with dose-reduction strategies ranging from no dose reduction, 300 mg daily, or 600 mg thrice weekly [4–6].

Encouraging outcomes with linezolid-based regimens for MDR/RR-TB have prompted interest in linezolid's use in regimens for RS-TB. However, multiple questions remain, including linezolid's efficacy in combination with other first line-drugs. Pharmacokinetic studies suggest that rifampin reduces linezolid AUC by 32% [16], but whether this interaction can be overcome via higher linezolid dosing is unknown [17]. Because TB requires multidrug treatment, it can be challenging to assess the contribution of an individual drug to treatment success. For MDR/RR-TB, an individual patient data meta-analysis found that patients with linezolid-containing regimens were more likely to achieve treatment success and less likely to die compared with those who did not receive linezolid [15]. Whether similar findings hold true for RS- TB is unknown.

It is unclear whether the rate of linezolid-related AEs will differ in US settings from those reported in TB clinical trials [18]. Most published experience has occurred outside of the US and in resource-limited settings where therapeutic drug monitoring is not available; trial participants were generally younger and had fewer medical comorbidities (other than HIV-1) than commonly seen in many US TB clinics. In the ZeNix trial, the median age was 36 years; 11% had diabetes, and 20% were people with HIV (PWH) [2]. In contrast, in 2024 in San Francisco the median age was 64 years, 21% had diabetes, and 2% were PWH [19]. Careful consideration is merited when using linezolid in older persons, given their greater risk of AEs to first line TB drugs [20, 21].

With growing use of BPaL/M, there is increased interest in developing AE monitoring strategies for linezolid given its narrow therapeutic index. Dose-adjustments based on serum linezolid troughs is recommended, as a value >2 μg/mL may predict toxicity [22]. Screening for symptoms of neuropathy and drop in hemoglobin (>10% in the first 4 weeks) may also be useful [23]. The optimal dosing strategy in these situations is unclear. Recommended approaches include reducing linezolid to 300 mg daily, though we favor dosing linezolid at 600 mg thrice weekly [11, 15]. Some experts recommend obtaining linezolid peaks to optimize dosing, although we do not routinely obtain peaks due to logistical challenges [24].

Two US case series have reported using linezolid (in the context of BPaL/M) in seven patients undergoing treatment for RS-TB complicated by rifampin-intolerance [25, 26]. Our study provides further evidence that linezolid may be a reasonable option as part of multi-drug therapy for RS-TB. We found that with close clinical and laboratory monitoring with dose adjustment, nearly two-thirds of our patients tolerated linezolid for at least 60 doses. Reassuringly, many were older, with a median age of 67 years. Moreover, immunocompromised patients were able to receive linezolid for a similar duration as immunocompetent persons. In contrast to the recent meta-analysis of three trials evaluating BPaL-containing regimens [18], none of our patients developed peripheral neuropathy, which may be due to close clinical monitoring, differences in linezolid exposure (eg, due to comorbidities such as chronic renal insufficiency, concomitant medications such as rifampin, population differences in linezolid metabolism, or treatment duration), use of linezolid trough levels, and clinicians’ low threshold to dose reduce or permanently discontinue linezolid.

Limitations of this report include its single-center retrospective design and probable variation in provider practice regarding dose reduction versus permanent discontinuation of linezolid. More studies are needed to delineate the role of linezolid for RS-TB and to develop practical dosing and monitoring strategies. Newer generation oxazolidinones which may be associated with fewer AEs should be further investigated [27–30].

## References

[1] Conradie F, Diacon AH, Ngubane N, et al Treatment of highly drug-resistant pulmonary Tuberculosis. N Engl J Med 2020; 382:893–902. 32130813 10.1056/NEJMoa1901814 PMC6955640

[2] Conradie F, Bagdasaryan TR, Borisov S, et al Bedaquiline-pretomanid-linezolid regimens for drug-resistant Tuberculosis. N Engl J Med 2022; 387:810–23. 36053506 10.1056/NEJMoa2119430 PMC9490302

[3] Nyang’wa B-T, Berry C, Kazounis E, et al A 24-week, all-oral regimen for rifampin-resistant Tuberculosis. N Engl J Med 2022; 387:2331–43. 36546625 10.1056/NEJMoa2117166

[4] Guglielmetti L, Khan U, Velásquez GE, et al Oral regimens for rifampin-resistant, fluoroquinolone-susceptible Tuberculosis. N Engl J Med 2025; 392:468–82. 39879593 10.1056/NEJMoa2400327 PMC7617355

[5] Conradie F, Badat T, Poswa A, et al BEAT Tuberculosis: a randomized controlled trial of a 6-month strategy for rifampicin-resistant tuberculosis. medRxiv 2025.05.04.25326549. 6 May 2025, preprint: not peer reviewed.

[6] Guglielmetti L, Khan U, Velásquez GE, et al Bedaquiline, delamanid, linezolid, and clofazimine for rifampicin-resistant and fluoroquinolone-resistant tuberculosis (endTB-Q): an open-label, multicentre, stratified, non-inferiority, randomised, controlled, phase 3 trial. Lancet Respir Med 2025; 13:809–20. 40683298 10.1016/S2213-2600(25)00194-8 PMC13508822

[7] Nuermberger E . Evolving strategies for dose optimization of linezolid for treatment of tuberculosis. Int J Tuberc Lung Dis 2016; 20:48–51. 28240573 10.5588/ijtld.16.0113

[8] MacGowan AP . Pharmacokinetic and pharmacodynamic profile of linezolid in healthy volunteers and patients with gram-positive infections. J Antimicrob Chemother 2003; 51:ii17–25. 12730139 10.1093/jac/dkg248

[9] Saukkonen JJ, Duarte R, Munsiff SS, et al Updates on the treatment of drug-susceptible and drug-resistant Tuberculosis: an official ATS/CDC/ERS/IDSA clinical practice guideline. Am J Respir Crit Care Med 2025; 211:15–33. 40693952 10.1164/rccm.202410-2096ST PMC11755361

[10] WHO consolidated guidelines on tuberculosis: module 4: treatment and care. Available at: https://www.who.int/publications/i/item/9789240107243. Accessed 20 August 2025. 40163610

[11] Chapter 4. Treatment | Curry International Tuberculosis Center. Available at: https://www.currytbcenter.ucsf.edu/products/page/chapter-4-treatment. Accessed 20 August 2025.

[12] Nahid P, Dorman SE, Alipanah N, et al Official American thoracic society/centers for disease control and prevention/Infectious Diseases Society of America clinical practice guidelines: treatment of drug-susceptible Tuberculosis. Clin Infect Dis 2016; 63:e147–95. 27516382 10.1093/cid/ciw376 PMC6590850

[13] CTCAE and AE Reporting—NCI. 2025. Available at: https://dctd.cancer.gov/research/ctep-trials/for-sites/adverse-events. Accessed 20 August 2025.

[14] 2020 Report of Verified Case of Tuberculosis (RVCT) Instruction Manual. 2021; Available at: https://www.cdc.gov/tb/media/pdfs/Report-of-Verified-Case-of-Tuberculosis-RVCT.pdf. Accessed 15 October 2025.

[15] Nahid P, Mase SR, Migliori GB, et al Treatment of drug-resistant Tuberculosis. An official ATS/CDC/ERS/IDSA clinical practice guideline. Am J Respir Crit Care Med 2019; 200:e93–142. 31729908 10.1164/rccm.201909-1874ST PMC6857485

[16] Linezolid prescribing information. 2013; Available at: https://www.accessdata.fda.gov/drugsatfda_docs/label/2014/021130s032,021131s026,021132s031lbl.pdf. Accessed 20 August 2025.

[17] Pea F, Viale P, Cojutti P, Del Pin B, Zamparini E, Furlanut M. Therapeutic drug monitoring may improve safety outcomes of long-term treatment with linezolid in adult patients. J Antimicrob Chemother 2012; 67:2034–42. 22553142 10.1093/jac/dks153

[18] Hasan T, Medcalf E, Nyang’wa B-T, et al The safety and tolerability of linezolid in novel short-course regimens containing bedaquiline, pretomanid, and linezolid to treat rifampicin-resistant Tuberculosis: an individual patient data meta-analysis. Clin Infect Dis 2024; 78:730–41. 37874021 10.1093/cid/ciad653 PMC10954324

[19] Tuberculosis in the City & County of San Francisco, 2024 | SF.gov. Available at: https://www.sf.gov/tuberculosis-in-the-city-county-of-san-francisco-2024. Accessed 20 August 2025.

[20] Louie JK, Keh C, Agraz-Lara R, Phillips A, Graves S. Adverse events associated with treatment for pan-susceptible Tuberculosis in San Francisco. Clin Infect Dis 2023; 76:1121–4. 36322073 10.1093/cid/ciac867

[21] Gardner Toren K, Spitters C, Pecha M, Bhattarai S, Horne DJ, Narita M. Tuberculosis in older adults: seattle and king county, Washington. Clin Infect Dis 2020; 70:1202–7. 30977788 10.1093/cid/ciz306

[22] Song T, Lee M, Jeon H-S, et al Linezolid trough concentrations correlate with mitochondrial toxicity-related adverse events in the treatment of chronic extensively drug-resistant Tuberculosis. EBioMedicine 2015; 2:1627–33. 26870788 10.1016/j.ebiom.2015.09.051 PMC4740314

[23] Imperial MZ, Nedelman JR, Conradie F, Savic RM. Proposed linezolid dosing strategies to minimize adverse events for treatment of extensively drug-resistant Tuberculosis. Clin Infect Dis 2022; 74:1736–47. 34604901 10.1093/cid/ciab699 PMC9155613

[24] Maranchick N, Peloquin CA, Haley CA. Linezolid dosing and pharmacokinetics in north American patients with Tuberculosis. Clin Infect Dis 2025; 81:830–7. 39834217 10.1093/cid/ciaf027

[25] Haley CA, Schechter MC, Ashkin D, et al Implementation of bedaquiline, pretomanid, and linezolid in the United States: experience using a novel all-oral treatment regimen for treatment of rifampin-resistant or rifampin-intolerant Tuberculosis disease. Clin Infect Dis 2023; 77:1053–62. 37249079 10.1093/cid/ciad312 PMC11001496

[26] Goswami ND, Ashkin D, Haley CA; BAM Project Team. Pretomanid in the treatment of patients with Tuberculosis in the United States. N Engl J Med 2022; 387:850–2. 36053513 10.1056/NEJMc2119461

[27] Chen RH, Burke A, Cho J-G, Alffenaar J-W, Davies Forsman L. New oxazolidinones for Tuberculosis: are novel treatments on the horizon? Pharmaceutics 2024; 16:818. 38931939 10.3390/pharmaceutics16060818 PMC11207443

[28] Heinrich N, Manyama C, Koele SE, et al Sutezolid in combination with bedaquiline, delamanid, and moxifloxacin for pulmonary tuberculosis (PanACEA-SUDOCU-01): a prospective, open-label, randomised, phase 2b dose-finding trial. Lancet Infect Dis 2025; 25:1208–18. 40645196 10.1016/S1473-3099(25)00213-0

[29] Minja LT, van der Feltz I, Manyama C, et al Delpazolid in combination with bedaquiline, delamanid, and moxifloxacin for pulmonary tuberculosis (PanACEA-DECODE-01): a prospective, randomised, open-label, phase 2b, dose-finding trial. Lancet Infect Dis 2025; 25:1219–29. 40645198 10.1016/S1473-3099(25)00289-0

[30] Harrison LJ, Velásquez GE, Kempker RR, et al ACTG a5409 (RAD-TB): study protocol for a phase 2 randomized, adaptive, dose-ranging, open-label trial of novel regimens for the treatment of pulmonary tuberculosis. Trials 2025; 26:291. 40817073 10.1186/s13063-025-08973-w PMC12357366

